# Clown-care reduces pain in children with cerebral palsy undergoing recurrent botulinum toxin injections- A quasi-randomized controlled crossover study

**DOI:** 10.1371/journal.pone.0175028

**Published:** 2017-04-17

**Authors:** Hilla Ben-Pazi, Avraham Cohen, Naama Kroyzer, Renana Lotem- Ophir, Yaakov Shvili, Gidon Winter, Lisa Deutsch, Yehuda Pollak

**Affiliations:** 1 Neuropediatric Unit, Shaare Zedek Medical Center, Jerusalem, Israel; 2 Department of Pediatrics, Shaare Zedek Medical Center, Jerusalem, Israel; 3 Biostatistical Consulting (L.D), BioStats, Modien, Israel; 4 School of Education, The Hebrew University of Jerusalem, Jerusalem, Israel; IRCCS E. Medea, ITALY

## Abstract

**Objective:**

We investigated the impact of clown-care on pain in 45 children with cerebral palsy who underwent recurrent Botulinum-toxin injections (age 7.04*±* 4.68 years). Participants were randomized to receive either clown (*n* = 20) or standard (*n* = 25) -care.

**Methods:**

Pain Visual-Analogue-Scale (range 1–5) was reported before and after procedures. Pain assessment was lower for children undergoing Botulinum-toxin injections with clown-care (2.89± 1.36) compared to standard-care (3.85± 1.39; *p* = 0.036) even though pain anticipated prior to procedures was similar (~3).

**Findings:**

Children who underwent the first procedure with clown-care reported lower pain even after they crossed-over to the following procedure which was standard (*p* = 0.048). Carryover effect was more prominent in injection-naïve children (*p* = 0.019) and during multiple procedures (*p* = 0.009). Prior pain experience correlated with pain in subsequent procedures only when first experience was standard-care (*p* = 0.001).

**Conclusions:**

Clown-care alleviated pain sensation during Botulinum-toxin injections and initial clown-care experience reduced pain during subsequent injections even though clowns were not present.

**Trial registration:**

clinicaltrials.gov ID # NCT01377883.

## Introduction

Children with cerebral palsy (CP), the most common cause of disability in children, are treated with recurrent Botulinum toxin (BTX) injections into affected muscles as one of the treatments of hypertonicity and improvement of motor function [1]. Pain, which anyway affects 70% of children with CP on a daily or weekly basis, can be augmented during physical and medical treatments [2, 3]. BTX injections are the gold standard treatment for focal hypertonia but typically repeated every few months to maintain the effect [4]. The procedure is short, but causes pain and anxiety [5] since needle-related procedures are a source of pain and distress and general anesthesia is, for the most part, avoided [6]. Other techniques of analgesia, such as light sedation, are standard of care in many institutions and include medications such as midazolam or inhaled nitrous oxide and non-pharmacological interventions [7–9]. Cochrane meta-analysis has demonstrated that certain non-pharmacological interventions help manage or reduce needle related pain [10–12], but we found no studies regarding the efficacy of non-pharmacological treatments for recurrent needle procedures.

Clown-care is safe and fun and is a rapidly developing field in medical care. Accumulating evidence indicates that it aids coping during difficult medical conditions in the hospital setting [13–17]. The use of medical clowns for needle-related procedures has been shown to alleviate pain in young children and reduce parental anxiety [18, 19]. We hypothesized that clown-care would have an impact on pain experience during recurrent BTX injections in children with CP and conducted a quasi-randomized-control, crossover study assessing pain in recurrent BTX injections with or without clowning.

## Methods

### Participants

**Fig 1:**
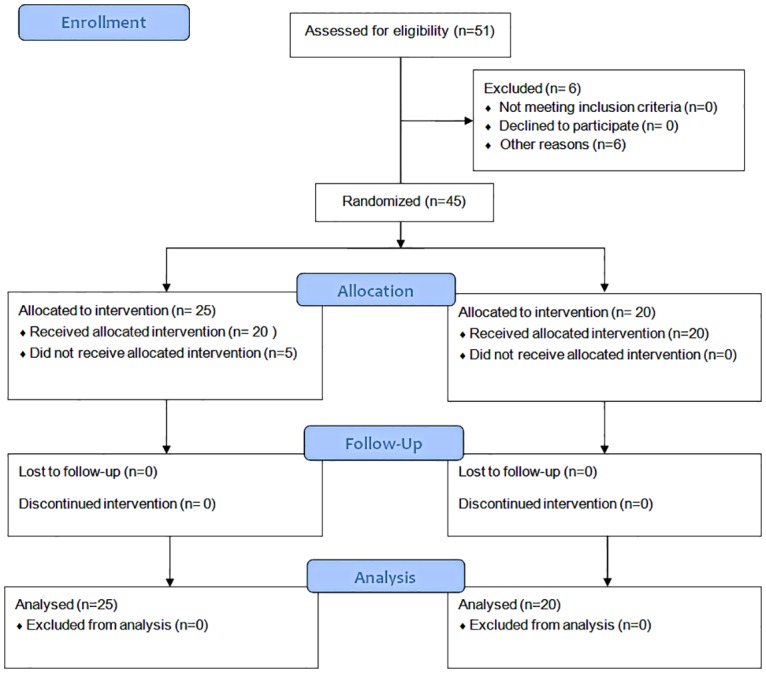
CONSORT flow diagram. Study design process: enrollment, randomization, Allocation, follow-up and analysis.

Children (*n* = 45; mean age 7.04 ± 4.68 years; 31 boys, 14 girls) with CP (mean gross motor functional classification scale (GMFCS) 2.86 ± 1.07) for whom BTX treatment was indicated were included in the study. Inclusion criteria: 1—children ages 1.5–18 years at first injection; 2—referral for BTX. Exclusion criteria: 1—children with extremely limited communication skills (such as severe mental retardation; *n* = 3); 2—autistic spectrum disorders (*n* = 2); 3—Severe anxiety requiring general anesthesia (*n* = 1; Fig 1). Two injectors (senior and fellow) performed the procedure. Muscles were localized using electromyogram (EMG) guidance without stimulation, adding background noise but not pain. The Helsinki committee at Shaare Zedek Medical Center approved the two parts of the study. The first part was a quasi-randomized controlled trial (April 2009; clinicaltrials.gov ID # NCT01377883), the second part was a crossover trial (April 2011). All parents signed a written informed consent form. The individuals in this manuscript gave written informed consent (as outlined in PLOS consent form) to publish their pictures.

### Quasi-randomized controlled trial (recruitment and intervention: May 2009–May 2014)

Randomization was performed according to the clowns’ schedule (usually covering the hospital's needs until noon). The parents and secretary scheduled the intervention appointment without knowing if clowning would be available. A child who had an appointment when clowns were available was allocated to the study group (*n* = 20), while the control group consisted of participants for whom clowns were unavailable (*n* = 25). None of the children were afraid or refused clown-care and it was not necessary to change the group allocation. One child requested that the clown remove his red nose. Power analysis revealed that such sample size could identify a difference of 0.76 standard deviations, with significance level of 0.05 and power of 0.8.

### Crossover trial (recruitment and intervention: June 2012–May 2014)

Children returning for a subsequent procedure were allocated to cross-over according to the randomization of their previous procedure (*n* = 25). Children who had previously received clown-care did not receive clowning and vice- versa. Power analysis revealed that such sample size will identify a medium effect size of f = 0.29, with 0.5 correlation between measures, a significance level of 0.05 and power of 0.8. Twenty children were not included in the crossover because recurrent BTX was not indicated (n = 14) or due to their request (clown, n = 2; standard, n = 4).

### Standard intervention

All children received the following:

#### Preparation and information

We explained the steps of the procedure according to age and cognitive abilities: EMG stickers, cleaning, cooling (ethyl chloride), needle insertion and importance of hearing the EMG.

#### Procedure

Children lay/sat with parent/s adjacent. BTX injections were performed under EMG guidance. Large muscles were injected twice to enhance diffusion. Children could see injections in upper limbs but not in lower limbs.

#### Memory change and positive reinforcement

Following the BTX injection, the medical staff present spoke to the child positively and offered prizes attempting to reframe the negative memories into more positive ones. In the control condition, an additional staff member assisted with technical aspects of the procedure, receiving no instructions regarding pain.

### Medical clowning procedure

**Fig 2:**
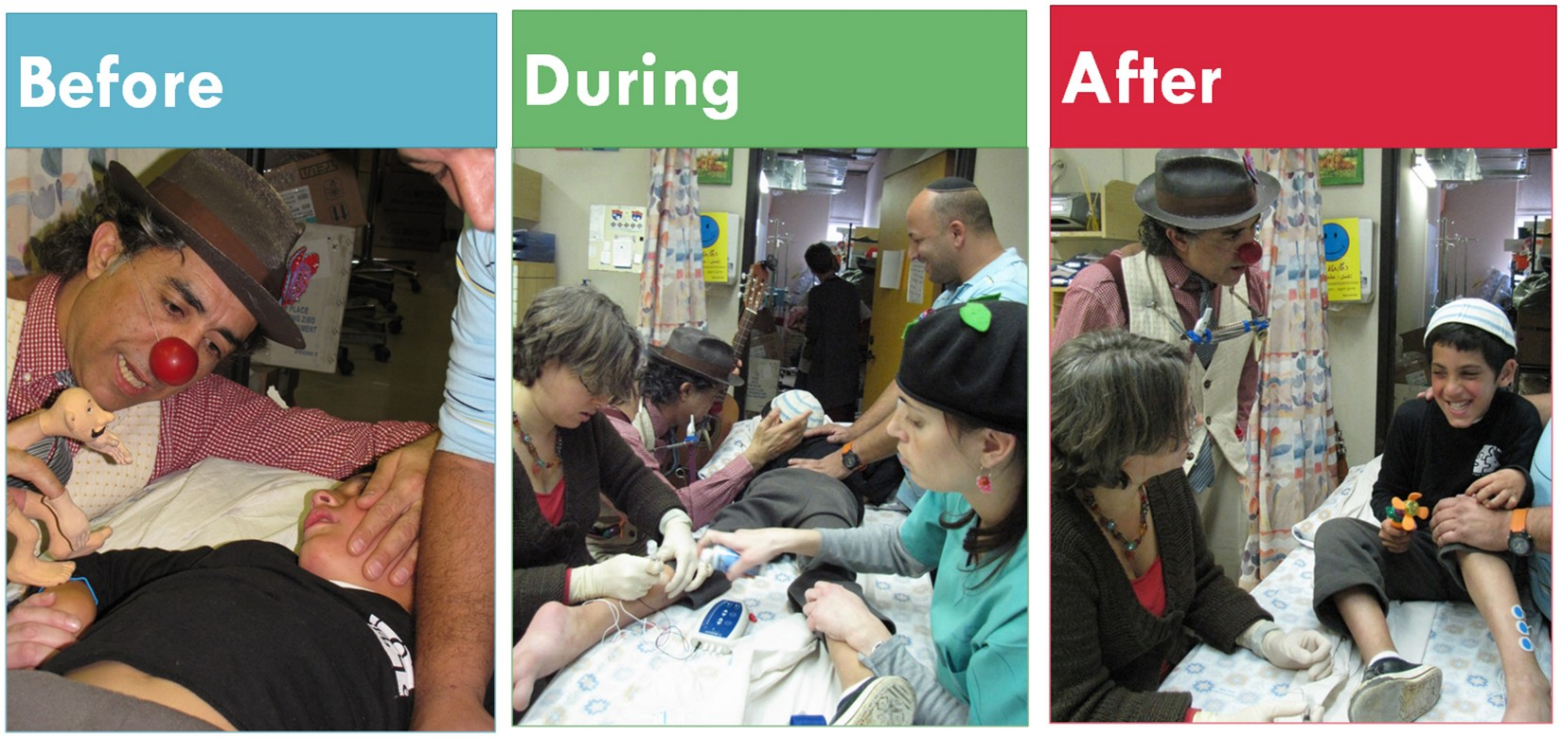
Medical clowning effect before, during and after BTX injections. Before: Clown engaging and distracting the anxious child who pays attention to the scene in front of him. During the injection: Clown and child in a secluded atmosphere at the other end of the table; parent watching with a smile. After: Child is laughing leaving the room empowered.

We used the services of professional, experienced medical clowns (previous stage experience, formal clown-care training and experience) in addition to the measures described in the standard intervention. The nursing staff welcomed the clown and there was routine cooperation with the day-care staff.BTX injection requirements were discussed with psychologists, clowns and physicians adjusting clowning techniques to the procedure and population. The medical clown was introduced before the injections and continued clowning until after the injection to enhance positive memory (Fig 2). Clowning strategies were chosen by the clown (according to the child’s age, cognitive level, character and preference) and were performed quietly so that EMG guidance could be heard. Examples of clowning strategies:

#### Cognitive coping

Encouraging a child to cope with the challenge. The clown or the child repeats positive thoughts (e.g., “You can do this”; “You are stronger than the needle”).

#### Guided imagination

Cognitive techniques used to encourage children's coping with the pain and distress by imagining a pleasant object or experience (e.g., a captain on a ship).

#### Empowerment

Children feel empowered by controlling the actions of the clown (e.g. the clown "falls" when the child gestures), emphasizing the strength and abilities of the child.

#### Emotional reflections

Clowns, sensing the state of the child, act it out in an exaggerated fashion "allowing" the child to react freely with unexaggerated feeling.

### Outcome measures

#### Pain

Degrees of pain associated with the procedure were measured. We used the Visual Analogue Scale (VAS) with a 5-face scale (from a very happy face = 1/5 to a very sad face = 5/5), considered to be a reliable tool in children, including those with CP [20–23]. The primary outcome measure was the degree of pain experienced and rated after the procedure ("Show on the ruler how painful were the injections?"; VAS-after). The second outcome measure was the perception of the expected pain as rated immediately prior to the injection, in the room before seeing the injection equipment ("Show on the ruler how painful do you think the injections will be?"; VAS-before). VAS-after of ≥4/5 was considered severe pain. Escorting parent reported pain for children who were unable to grade pain (i.e. children under 5 years or with significant intellectual disability), using the same VAS ruler [21, 24].

#### Effectiveness questionnaires

Short structured questionnaires (using a 1–5 scale) were used to assess effectiveness of the clown care as perceived by the parents and the healthcare staff. The questionnaires were completed immediately following the intervention. Parents were asked to report the effectiveness of the clown care on the child and on the parent. Clowns recorded the technique used and how effective was the intervention. Nurses were asked to record the difficulty managing/holding the child and listening to the EMG device. Physicians recorded the level of difficulty in targeting the muscle and listening to the EMG device. We attempted to use gait assessment (10 meter) before and after intervention but we realized that it was very dependent on the child's mood and was not used for analysis.

Statistical analyses were performed using SAS V9.3 (SAS Institute, Cary, NC, USA). Groups were compared depending on data type with t-tests, ANOVA, chi-squared test or Fisher’s exact test when relevant. Correlation between two continuous variables was assessed with Pearson correlation coefficients. The effect of VAS-before, age, gender, previous BTX injections, anatomical distribution of injections, number of muscles injected, injection of deep muscles, amount of BTX, cognitive and language level on the difference between the groups was evaluated with regression models. Repeated measures ANOVA (RMA) were performed to assess the crossover data of the study and also the longitudinal data. A p-value of 0.05 or lower was considered statistically significant.

## Results

### Quasi-randomized control trial (*n* = 45; Table 1)

**Table 1:** Clinical characteristics.

Parameter	Total (n = 45)	Clown care (n = 20)	Standard care (n = 25)	P value
Age	7.04 ± 4.66 y	7.81 ± 4.70 y	6.40 ± 4.67 y	0.3272
Gender M:F	31:14	15:5	16:9	0.5255
GMFCS	2.86 ± 1.07	2.86 ± 1.15	2.85 ± 1.00	0.9756
Anatomical distribution				0.0849
Diplegia	16	11 (55%)	5 (20%)	
Hemiplegia	13	4 (20%)	9 (36%)	
Quadriplegia	15	5 (25%)	10 (40%)	
Triplegia	1	0	1 (4%)	
School attendance				0.5255
Main stream	14 (31%)	5 (25%)	9 (36%)	
Special education	31 (69%)	15(75%)	16 (64%)	
Communication skills				0.7310
Verbal	35 (78%)	15 (75%)	20 (80%)	
Non- verbal	10 (22%)	5 (25%)	5 (20%)	
# muscles	3.0 ± 1.6	3.4 ± 1.7	2.6 ± 1.3	0.1248
BTX amount (IU)		179 ± 85	162 ± 121	0.6044
Upper Limb	14	7 (35%)	7 (30%)	0.7500
Lower Limb	35	17 (85%)	18 (78%)	0.7041
Report				0.6337
Child	19	10 (53%)	9 (45%)	
Parent	20	9 (47%)	11 (55%)	
Injector				0.4967
Specialist	37	19 (76%)	18 (90%)	
Fellow	3	2 (8%)	1 (5%)	

**Fig 3:**
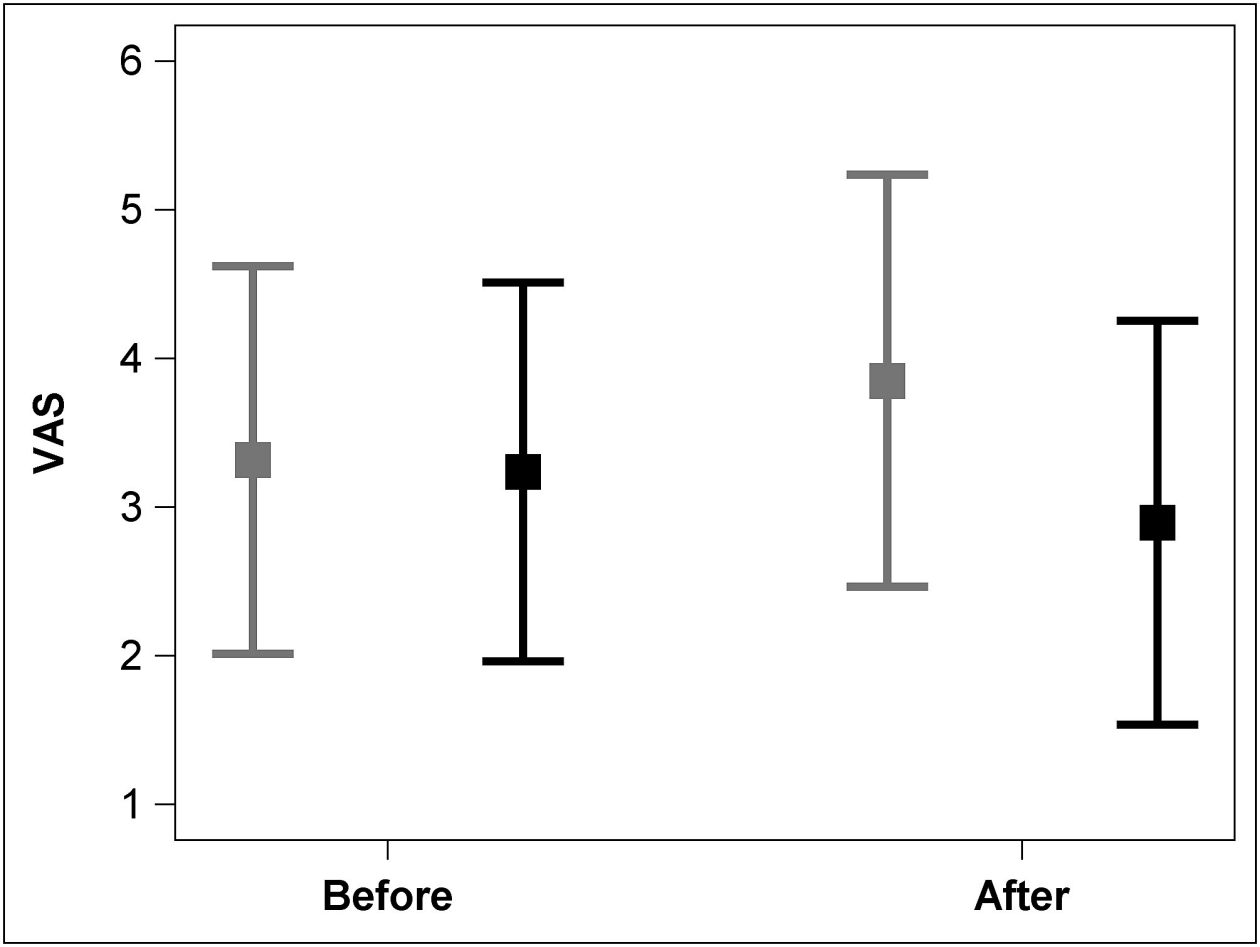
Pain (Visual Analogue Scale) after BTX injections with and without clown-care. Box plots (diamond = mean, solid line in center = median) of pain after the first procedure according to the Visual Analogue Scale (VAS; range 1 = no pain to 5 = severe pain): After the procedure the pain was experienced as moderate for the clown group (black) and severe in controls (grey).

Pain (VAS-after) was lower with clown-care: Children with CP who received BTX with medical clowns reported less pain following the procedure (VAS-after 2.89 ± 1.36) compared to the children who received BTX with standard care (VAS-after 3.85 ± 1.39; *2-tailed-t-test*, *p* = 0.036). Prior to the procedure, there was no difference between pain rating by the study group and controls (VAS-before 3.24 ± 1.27 and 3.32 ± 1.30, respectively; *2-tailed-t-test*, *p* = 0.85) even though children met with the clowns before the intervention (Fig 3). The effect size of clown-care on pain assessment after the procedure was moderate (*Cohen’s d* = 0.71) and low before it (*Cohen’s d* = 0.06).

Children without clowning experienced severe pain: Most (14/20; 70%) children who underwent BTX injection with the standard protocol reported severe pain (VAS-after≥4), while the majority (13/19; 68%) of children receiving clown-care reported pain that was not severe (VAS-after<4; *Fisher's exact test*, *p* = 0.026).

The experienced pain was lower than expected with clown-care and was greater with standard care: Pain report changed in most cases (27/42; 64%) before and after the procedure. While children who received clown-care reported a reduction in pain (VAS before- VAS after; mean -0.34 ± 1.58, N = 19), children who received standard care reported greater pain (mean +0.74 ± 1.78;, N = 19).

Clinical characteristics that did not impact pain, nor were found prognostic for pain after the injection: Gender, GMFCS, school attendance, anatomical distribution, communication skills, injection number, number of muscles injected, upper or lower limbs, BTX amount, VAS-before, injector proficiency (specialist vs fellow) and sensitive sites (*multivariate ANOVA; p* = 0.29–0.88). Child age was found statistically significant in the model (*p* = 0.035), with younger children reporting higher pain. This may be due to the fact that it was the parents who reported VAS pain scores for the younger children, and parents had a tendency (*p* = 0.09) to give higher VAS scores irrespective of group (S1 Table).

### Crossover trial (*n* = 25 (18 injection naïve); age 6.83 ± 4.42 years, 18:7, M:F)

**Fig 4:**
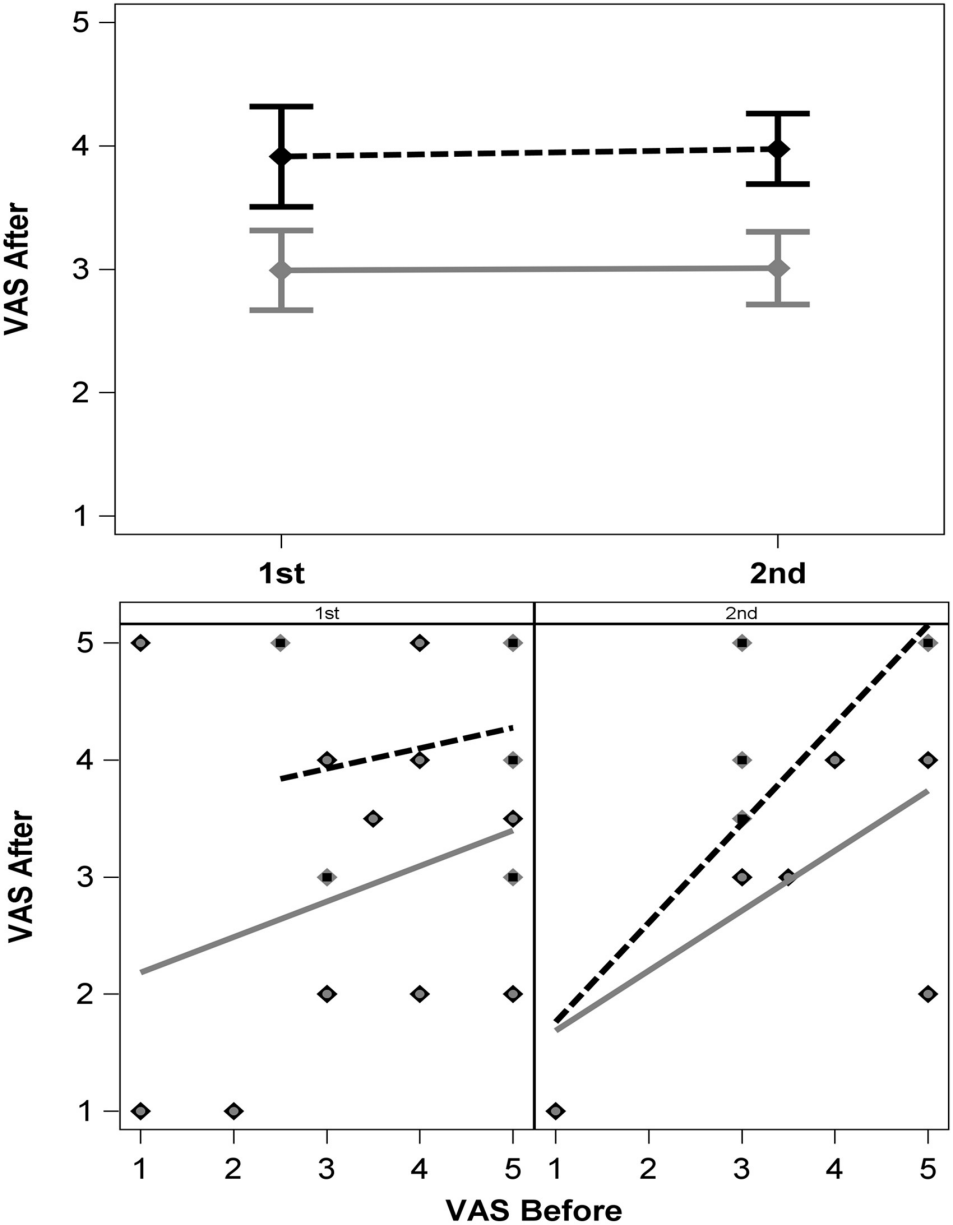
Carry over effect—Pain levels remained constant despite crossover (with/without clowning). (A) Pain levels (VAS-after LSmean ±SE) remained stable in each group (grey solid line: clowning 1^st^-> standard 2^nd^; black dashed line: standard 1^st^-> clowning 2^nd^) and did not change between the first and second procedure. However, pain was lower for children who received clowning during the first injection (grey) compared to those who received clowning only on the second injection (black). (B) Anticipated pain (before) did not correlate with experienced pain (after) the first procedure for both groups. (C) However, pain experience (after) correlated with anticipated pain (before) of the second injection for those who received standard procedure in the first time (black) but not for those who previously received clowning (grey).

Clowning had an impact on both the first and second procedure (carryover effect): The child who was exposed to clown-care on the first injection reported less pain (VAS-after 2.84 ± 1.38) on the second injection compared to those who received standard care for their first procedure (4.11± 0.93; *2-tailed t-test*, *p* = 0.032). Pain levels did not change from the first to the second procedure and were significantly lower in children who received clown care first (*ANOVA*, *p* = 0.048) compared to children who received the standard procedure first (Fig 4). Carryover effect was more prominent in children who were injection naïve *(n* = 18; *ANOVA*, *p* = 0.019). The pain expected before the procedure (VAS-before 1^st^ vs VAS-before 2^nd^) was similar in both groups (*Repeated Measures ANOVA*, *p* = 0.274) whether the child was injection naïve or not (*p* = 0.20).

Clowning carryover effect was mainly reported by children and not parents: For children who received the clowning during the first procedure pain levels were reported as lower in the second procedure to a greater extent when children reported pain themselves (VAS- after-2^nd^ = 2.0) compared to parental report (3.53 respectively, *p* = 0.003). There was no difference between the children's and parents' report when the first procedure was standard and clowning was given at the second procedure (VAS-after-2^nd^; *p* = 0.89).

No other parameters had an carryover effect: None of the other variables (age, gender, GMFCS, school attendance (main stream vs special education), CP type, verbal abilities (verbal/non-verbal = uses language/gestures as main mode communication), number of muscles injected, injector proficiency, sensitive sites) was found to impact pain after the procedures, whether first or second (VAS-after).

### Longitudinal arm (n = 25)

**Fig 5:**
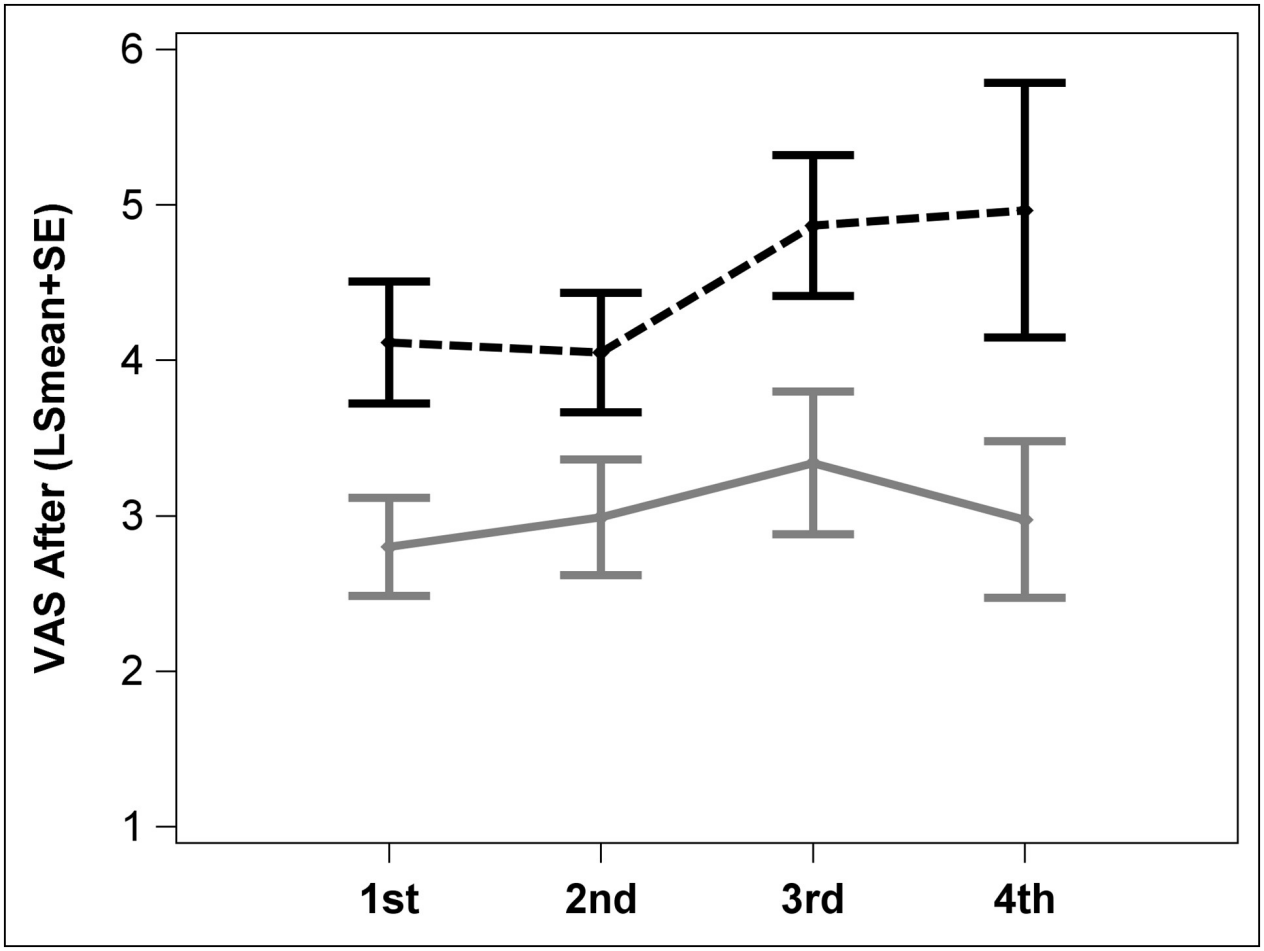
Pain as a function of clown presence during first procedure (longitudinal). Pain levels (VAS-after LSmean ± SE) were lower for the children receiving clown care during the first injection (grey solid line) and remained low (VAS<4) during second, third and fourth injections. Pain was high (VAS>4) for children who did not receive clown care during the first injections.

Once we observed that previous injection experience is important, we checked the influence of the first injection beyond the crossover trial. Twenty five children whose first injection experience ever was with a clown (N = 15) or without a clown (N = 10) were evaluated in this arm, we excluded children who had their first injection before enrolling in the study from this analysis. In children where the first procedure was performed with clowning 7 had only one injection performed and the remainder 2 or more (median = 2 range 1–5 injections; median interval between two procedures 0.5; range 0–1.5 years). All children who experienced the standard procedure first had 2 or more injections (median = 2.5 range 2–4 injections; median interval between two procedures 0.5; range 0–3 years). Children who experienced their first BTX with clowning reported lower pain for up to 4 recurrent injections compared to children who received standard care during the first BTX injection (*Repeated measures ANOVA*; p = 0.0139, Fig 5).

### Effectiveness questionnaires

Questionnaires were available for 24 children in the standard procedure and 16 during the clowning. There was some difficulty holding the child (~2/5) in both groups as reported by the nurse (clowning 2.3, *SD* 0.8 and 2.2, SD1.0; *2-tailed- t-test*, *p* = 0.817). In both groups there was a problem hearing the EMG (90%, *SD* 18% of muscles were heard in the standard and 77%, *SD* 29% were heard in the standard procedure; *p* = 0.144 physician). Clowning and standard procedures were both regarded as moderately effective (~2.4/5) by parents (2.3, *SD* 1.5 and 2.6, *SD* 1.5 respectively; *p* = 0.736). Due to the small sample size of reports after the first injection (*n* = 14), we could not analyze these parameters for injection in naïve children. Clowning strategies as reported by the clowns after the procedure were variable: distraction and music were the most common techniques reported (11/16, 69%) followed by empowerment (9/16, 56%).

## Discussion

We found that clown-care is an effective method in reducing pain perception following recurrent BTX injections in children with CP. Most children receiving clown-care reported lower pain levels similar to other clown studies,[25] which is the goal of behavioral and cognitive interventions [20]. Children who did not receive clown care experienced higher levels of pain during the first injection than was anticipated, whereas children receiving clown-care therapy experienced pain equal to or lower than anticipated. This finding was consistent across subsequent injections. This indicates that clown-care can alleviate acute pain and creates resilience for future procedures. Although the clown interaction began before the procedure, it did not relieve anticipated pain before the first experience, indicating that clown care is effective when conducted during the needle procedure itself, not before.

First experience was found to crucially affect subsequent injections. Apparently if a child had a specific experience, it was difficult to alter the perceptions, as shown previously in children younger than 8 years [26]. This observation suggests that children are capable of developing memories of pain that can influence their subsequent reactions to similar experiences [27, 28]. Clowning may impact the experience of the first procedure in a purely physiological way. It has been demonstrated that social laughter elevates pain threshold, which provides a possible explanation for the pain difference between the clown-care and control group [29]. Physiological effects of clowning were shown in a randomized control study reporting increased conception following *in vitro* fertilization procedures with clown-care [30]. The altered pain memory had a carry-over effect to other procedures similar to other pain treatments; effective pain medication alleviates pain in subsequent episodes [31, 32]. However, if the procedure was experienced as traumatic, clowning could not alter this.

A clowning study similar to ours did not find that clown-care shortened the period of crying during BTX injections [33]. This study documented the effect of clowning by the period of crying rather than a valid pain assessment tool [34, 35]. Moreover, our clowning techniques did not include painted faces and thus is considered less frightening. Moreover, the clowning effect over time was not assessed.

Clinicians are not always cognizant of the pain and trauma experienced by children when undergoing interventions. Often the concern about pain is dismissed with the maxim "procedures without anesthesia reduce medical risks". Most BTX procedures are performed without general anesthesia for this reason, [6] and sedation is not always available; in these cases medical clowns are able to alleviate pain and reduce subsequent trauma. The opportunity to modulate pain impact during the first procedure has a significant carryover effect. Painful procedures that compromise the child's well being are part and parcel of medical care in those with physical disabilities. The need to improve the quality of life in children with CP is extremely important, justifying the attention given to reduce the child’s discomfort without increasing risk. Our results indicate that clown-care during needle procedures reduce pain perception which hopefully will lead to better medical results and reduce the need for general anesthesia in children during medical examinations that require the cooperation of the child.

### Limitations of the study

The study used a quasi-randomized assignment and small sample size. The possible consequences for quasi-randomization is that there may have been a biased allocation and biasing of the group comparison due to lack of allocation concealment. We did not control for previous needle procedures (such as IV insertion), operations or painful treatments in the past or parental anxiety that may have impacted pain perception. Pain was recorded according to subjective reports since there is no known objective measure. The effect of clowning is assumed to occur by raising the child's pain threshold, but since the parents and staff could not be blinded to the procedure, clowning may have had an indirect impact on *the child by effecting the environment*. Pain reports were attained from parent or child, thus parent reported pain data are mixed with children reported pain data [36].

### In conclusion

Children with CP benefit from clown-care during Botulinum toxin injections. Clowning during the first encounter reduces pain during the current and subsequent procedures. This safe and novel alternative intervention during needle related procedures may provide better pain management in children with disabilities.

## Supporting information

S1 Consort Checklist (DOC)

S1 Protocol (DOC)

S1 Table Raw data spreadsheet. This spreadsheet includes all data used for analysis. https://figshare.com/articles/clown_data_xls/4802959.
(XLS)
