# Supplementary material for: Clown-care reduces pain in children with cerebral palsy undergoing recurrent botulinum toxin injections- A quasi-randomized controlled crossover study
Source: PLoS One. 2017 Apr 17;12(4):e0175028. doi: 10.1371/journal.pone.0175028 (PMC5393564; doi:10.1371/journal.pone.0175028)
Supplement: S1 Protocol — (DOC) [file pone.0175028.s002.doc]

**Does clown care contribute to the effectiveness of botulinum toxin**

**in children with cerebral palsy- a crossover study**

Submitted by: Hilla Ben-Pazi,MD, Neuropediatric Unit, Shaare Zedek Medical Center, POB 3235, Jerusalem 91031, Israel. Tel: 972-2-666-6641. Fax: 972-2-6555672. email: Benpazi@gmail.com

**Abbreviations**

BTX Botulinum toxin

CP Cerebral Palsy

VAS Visual Analogue Scale

EMG Electromyogram

**Abstract**

Children with cerebral palsy (CP) undergo multiple painful procedures such as Botulinum toxin (BTX) injections that are administered several times a year to improve motor function. While clown care reduces pain during BTX injections, its impact on the motor outcome has not been assessed. We hypothesize that clown care increases the effectiveness of the treatment and anticipate that better pain control during injection to the lower limb muscles will lead to a greater gait improvement. As the walking abilities are variable in children with CP, this study will be conducted as a crossover study*.*

We will enroll 40 ambulatory children with CP referred for BTX injections (age 3-18) to the lower limbs. Each child will be randomly assigned to receive either clown care (study) or standard care (control) intervention. Subsequently the second injection will be in a crossover manner (Group A: study-control; Group B: control-study). The main outcome measure will be the percent of improvement in casual gait, measured by a timed 10 meter test. We will also document the child's pain using the Visual Analogue Scale and assess the effectiveness of treatment by all adults present during the procedure using structured questionnaires. Gait improvement following clown care will be compared to the improvement after standard care. We will correct for covariant such as BTX dose, # of muscles injected, age, functional level, amount of physical treatments sessions and injection order. The secondary outcome measure will be the correlation between the gait improvement, pain reduction and the effectiveness of the procedure, as reported by the child, parent, nurse, clown and doctors. We hope that this novel study will address the challenging question regarding the impact of clowning on medical treatment outcome.

**1. Working hypothesis**

**1.1 Hypothesis:** BTX injections are performed under EMG guidance to target the motor point of the muscle injected by listening to the muscular activity. Since clown care reduces pain during the BTX injections in children with CP, it may also increase the cooperation of the child, thus enhancing the ability to target the motor point during the painful procedure. Injection technique is expected to impact on the motor outcome. We hypothesize that better pain control achieved with clown care will improve motor outcome of children receiving BTX injections with CP and anticipate that better pain control will lead to a greater functional improvement.

**1.2 Objectives:**

1. To compare gait improvement following BTX injection to the lower limb with and without clown care.
2. To check correlation between pain reduction reported by the child and gait improvement.
3. To check the correlation between pain reduction (reported by the child) and the clown effectiveness assessment (reported by clown, doctor, nurse and parent).

**2. Scientific Background**

Cerebral palsy (CP) consists of a heterogeneous group of non-progressive clinical syndromes characterized by motor and postural impairments, resulting from an insult to the developing brain. CP is the most common (2:1000 live births) cause of disability in children and is characterized by signs of hypertonia and muscle weakness.[^1^](#_ENREF_1) Treatments for CP are directed at maximizing the quality of life by improvement in daily function and reduction of the extent of disability.[^2^](#_ENREF_2) Seventy percent of children with CP experience recurrent chronic pain of moderate intensity on a daily or weekly basis which is augmented during medical procedures.[^3^](#_ENREF_3)'[^4^](#_ENREF_4) Many children undergo Botulinum toxin (BTX) injections into affected muscles. This procedure reduces spasticity, albeit temporarily and must be repeated every few months to maintain the effect. The procedure is short, but causes pain and anxiety[^5^](#_ENREF_5) since children in most centers do not undergo general anaesthesia.[^6^](#_ENREF_6) EMG guidance during the procedure increases XXX and requires cooperation of the patient (REF).

Needle-related procedures are a common source of pain and distress for children and parents. A systematic Cochrane meta-analysis has demonstrated that several cognitive-behavioral interventions help manage or reduce pain and distress; clowning was not included in the review.[^7^](#_ENREF_7) Clowning is safe and fun and is a rapidly developing field in medical care with evidence indicating that it aids when coping with difficult medical conditions in the hospital setting. We found that clown care is an effective method in reducing pain perception following BTX injections in children with CP. In our previous study none of the children receiving clown care reported severe pain (unpublished data, submitted for publication). While medical clowns were shown to reduce pain during BTX injections the effect of medical clowns on the outcome of painful procedures had not been studied.

We hypothesized that clown care would increase the child's cooperation during BTX injections, enabling better muscle targeting that will ultimately result in an enhanced motor outcome. To test this hypothesis, we will compare gait improvement *(ten meter test)* in children with CP treated with BTX injections with and without clowning and check its correlation with pin reduction using the Visual Analogue Scale (VAS)[^7^](#_ENREF_7) and clown care effectiveness.

**3. Methods**

**3.1 Participants:** Forty children will enroll in this study. Since we expect a 20% drop out we will recruit 50 children ages 2-18 year with CP for whom BTX injections to the lower limbs is indicated for improving gait. Children with minimal communication skills, autistic spectrum disorders will be a priori excluded. Data on gross motor functional classification scale (GFMCS), anatomical distribution of lesions, communication abilities and school system assignment (roughly indicative of cognitive level) will be collected. We will document the total amount of BTX used, which limbs were injected and the number of muscles treated during each procedure. Each Parent will sign an informed consent. The parent will be asked not to reveal to the child the reason for the study in order to minimize the child's bias.

**3.2 Study design:** This study will be structured as a crossover controlled study. Participants will be randomized into 2 groups: group A will receive medical clown intervention first and standard intervention second; group B will receive standard intervention first and medical clown intervention.

***3.2.1. Standard intervention*:** reflecting the regular intervention in the day care during needle procedures.

*Preparation and information:* To prepare the parent and child for the injection, the doctor and nurse will explain the steps of the procedure: placing electromyogram (EMG) electrodes, wiping the area with an alcohol swab, cooling with ethyl chloride, needle insertion into the muscle and the importance of EMG noise. The child will practice specific voluntary movements of affected muscles if needed to be contracted during the injection.

*Injection:* During the procedure, the child will be lying with the parent sitting next to him/her. The BTX injections will perform under EMG guidance. Two sites of injection per muscle are used to enhance diffusion. The child can not see the procedure during lower limb injections.

*Memory change and positive reinforcement:* Following the BTX injection, the medical staff present will speak to the child positively and offer a prize. This step is included to help the child reframe the negative memories of the procedure into more positive ones. This is standard procedure in the day care unit although its efficacy has not been studied.

*Volunteer attendance:* In the daycare unit there are young volunteers routinely present, assisting with technical aspects of the procedure. The volunteer is a part of the standard session, receiving no particular instructions in relation to the child's potential pain during the procedure.

***3.2.2. Medical clowning procedure:*** The medical clown will be introduced to the child and parents prior to the injections. The clowning technique during the procedure will be chosen by the clown according to the child’s age, cognitive level, character and preference and recorded. Each intervention will be performed quietly so that EMG guidance could be properly heard. Four techniques are commonly used:

*Cognitive coping:* encouraging a child to cope with the challenge. The clown or the child repeats a set of positive thoughts to energize the child during the procedure (e.g., “You can do this”; “You are stronger than the needle”).

*Imagery: a* cognitive technique used to encourage the child to cope with the pain and distress of the procedure by imagining a pleasant object or experience (e.g., enchanted forest) using an object such as a puppet or a fake needle.

*Empowerment*: the child is made to feel empowered by controlling the actions of the clown (e.g. the clown "falls" when the child touches his nose) emphasizing the strength and abilities of the child.

*Reflecting emotions:* the clown, sensing the state of the child, plays it out in an exaggerated fashion. The clown acts out the child's emotion that, "allows" the child to react freely, leaving the child stronger.

The clown will stay with the child, continuing the game until after the injection. The goal of the technique is that the child perceives him/herself as stronger than the clown and empowered from the experience (Figure 1).

**3.3 Outcome measures:** We will be assessing the child's pain, the effectiveness of the procedure and the improvement in gait by the following measures;

***3.3.1 Pain: Visual Analogue Scale (VAS)*** with a 5- face scale (from a very happy face to a very sad face), considered to be reliable tool for use in children for their and their parents’ ratings.[^10^](#_ENREF_10) The child will be asked to complete the VAS before and at the end of the procedure and grade the pain from 1-5; ranging from 1/5 (no pain) to 5/5 (extreme pain). If the child will be unable to grade pain (i.e. low cognitive function), the escorting parent will do the rating.[^10^](#_ENREF_10) The VAS will be first given before the child or parent sees the injection equipment and the second after receiving a tangible reward.

***3.3.2. Effectiveness: structured questionnaire:*** there will be a short written report to be completed by each individual. We will request participants not to share their assessments in order to minimize bias. Each adult attending the procedure will answer a short 1-5 scale reporting different aspects of the injection:

The parents will report the effectiveness that the clown had on the child and on the parent.

The clown will record the technique used and how effective was the intervention.

The nurse will record the difficulty managing/holding the child and listening to the EMG device.

The doctor will record the level of difficulty in targeting the muscle and listening to the EMG device.

***3.3.3.Gait: Timed 10 meter test****:* A 10 meter course will be marked with tape on the floor. The child will be instructed by the clown to walk at a comfortable rate 3 times. Then the child will be instructed to do walk again, but this time as fast as possible. This will be repeated 3 times. All tests will be recorded on video. The average comfortable and fast walking will be averaged and converted to m/min. [^11^](#_ENREF_11) We will compare the difference in the child's pace before the procedure and 3 weeks after the procedure at the time of maximal BTX effect. Gait improvement will be calculated as the percent of improvement (100*[speed after- speed before]/ speed before). All gait tests will be recorded for further evaluation if needed. The doctor evaluating the child before and after the procedure (parental interview, physical examination of spasticity and range of motion) will remain blinded to the timed 10 meter test.

**3.4. Data collection and analysis:** Pain score, effectiveness assessment and gait improvement will be compared between the standard and clown care intervention. Each child will serve as his own control. We will check if there was a greater improvement in gait after the clown care procedure versus the standard care. We will also check if the amount of improvement is correlated with pain score and effectiveness assessment.

The data will be analyzed with respect to the following background data parameters: age, gender, previous BTX injections, anatomical distribution of injections, number of muscles injected, amount of BTX, cognitive and language level.

Statistical analysis of background data and outcome measures will be performed using SSPS (IBM®, Statistics version 19); t-tests, ANOVA and chi-square test will be used to analyze differences between interventions. Relations between 2 continuous measures will be analyzed by Pearson correlation. For all statistical tests, the level of significance will be set at .05.

**5. Project staffing and management**

**Hilla Ben-Pazi, MD,** (PI) Neuropediatric Unit, Shaare Zedek Medical Center, Jerusalem, Israel. Dr Ben-Pazi is a pediatric neurologist with over a 7 years experience with BTX injections and is in charge of the movement disorders clinic at Shaare Zedek Medica Center: she will recruit patients and will be performing the BTX injection.

**Avraham Cohen, BA,** (CI) Department of Pediatrics, Shaare Zedek Medical Center, Jerusalem, Israel. Mr Cohen, an experienced medical clown, who was involved in our previous BTX study, will be in charge of the medical clowning intervention and assessment of 10 meter gait.

**Yehuda Pollak, PhD,** (CI) Neuropediatric Unit, Shaare Zedek Medical Center, Jerusalem, Israel. Dr Pollak has 9 years of experience as a research neuro-psychologist and is involved in most studies performed in the neuropediatric unit including our previous BTX study. He will assist with structuring the questionnaires and statistical analysis.

**Nava Badichi, BA**, Department of Pediatrics, Shaare Zedek Medical Center, Jerusalem, Israel. Ms Badichi, an experienced research assistant at the neuropediatric unit. She will coordinate the clinical procedures and record data for analysis. Ms Badichi will be the coordinator of the study: will perform the randomization, schedule appointment, collecting questionnaires and gait measures, recording the results of a data sheet for further statistical analysis, thereby minimizing transfer of information between researchers.

**References:**

**1.** Hagberg B, Hagberg G, Olow I, von Wendt L. The changing panorama of cerebral palsy in Sweden. VII. Prevalence and origin in the birth year period 1987-90. *Acta Paediatr* 1996; **85**: 954-60.

**2.** Mihaylov SI, Jarvis SN, Colver AF, Beresford B. Identification and description of environmental factors that influence participation of children with cerebral palsy. *Dev Med Child Neurol* 2004; **46**: 299-304.

**3.** Engel JM, Petrina TJ, Dudgeon BJ, McKearnan KA. Cerebral palsy and chronic pain: a descriptive study of children and adolescents. *Phys Occup Ther Pediatr* 2005; **25**: 73-84.

**4.** Castle K, Imms C, Howie L. Being in pain: a phenomenological study of young people with cerebral palsy. *Dev Med Child Neurol* 2007; **49**: 445-9.

**5.** Sanger TD. Hypertonia in children: how and when to treat. *Curr Treat Options Neurol* 2005; **7**: 427-39.

**6.** Bakheit AM. Botulinum toxin in the management of childhood muscle spasticity: comparison of clinical practice of 17 treatment centres. *Eur J Neurol* 2003; **10**: 415-9.

**7.** Uman LS, Chambers CT, McGrath PJ, Kisely S. Psychological interventions for needle-related procedural pain and distress in children and adolescents. *Cochrane Database Syst Rev* 2006: CD005179.

**8.** Dunbar RIM, Baron R, Frangou A, et al. Social laughter is correlated with an elevated pain threshold. *Proceedings of the Royal Society B: Biological Sciences* 2012; **279**: 1161-7.

**9.** Friedler S, Glasser S, Azani L, et al. The effect of medical clowning on pregnancy rates after in vitro fertilization and embryo transfer. *Fertil Steril* 2011; **95**: 2127-30.

**10.** Uman LS, Chambers CT, McGrath PJ, Kisely S. A systematic review of randomized controlled trials examining psychological interventions for needle-related procedural pain and distress in children and adolescents: an abbreviated cochrane review. *J Pediatr Psychol* 2008; **33**: 842-54.

**11.** Begnoche DM, Pitetti KH. Effects of traditional treatment and partial body weight treadmill training on the motor skills of children with spastic cerebral palsy. A pilot study. *Pediatr Phys Ther* 2007; **19**: 11-9.

Table 1 Clinical Characteristics

| **Background Data** | | | | **Procedure** | | | | | | **Pain assessment** | | | |
| --- | --- | --- | --- | --- | --- | --- | --- | --- | --- | --- | --- | --- | --- |
| **Age,**  **gender** | **GMFCS,**  **plegia** | **School** | **Comm.** | **Inject #** | **# muscles** | **Limb** | **Sensitive** | **Muscles** | **IU** | **Before** | **After** | **Before –**  **After** | **report** |
| **Botulinum Toxin Injections with standard care** | | | | | |  |  |  |  |  |  |  |  |
| 2,F | 5,Q | SE | NV | 1 | 2 | Low | No | ADDs | 140 | 3 | 4 | 1 | Parent |
| 3,M | 2,H | N | V | 1 | 2 | Low | Yes | GAS TP | 100 | 3 | 5 | 2 | Parent |
| 3,M | 2,H | N | V | 1 | 2 | Up | No | FCU PT | 40 | 5 | 4 | -1 | Parent |
| 3,M | 3,H | N | V | 1 | 2 | Up, Low | No | GAS PT | 60 | 4 | 4 | 0 | Parent |
| 3,M | 4,Q | SE | NV | 3 | 4 | Low | No | ADDs HAMs | 160 | 5 | 5 | 0 | Parent |
| 3,M | 2,D | SE | V | 1 | 2 | Low | No | GASs | 100 | 3 | 5 | 2 | Child |
| 5,M | 4,Q | SE | V | 1 | 4 | Low | No | GAS HAMs | 200 | 3.5 | 4 | 0.5 | Parent |
| 5,M | 2,H | SE | V | 3 | 2 | Low | Yes | GAS TP | 100 | 1 | 5 | 4 | Child |
| 6,M | 5,Q | SE | NV | 1 | 4 | Low | No | ADDs HAMs | 300 | 4 | 4 | 0 | Parent |
| 7,F | 4,Q | SE | V | 1 | 3 | Up | No | PT,BIC,BR | 80 | 1 | 4 | 3 | Child |
| 7,M | 2,D | N | V | 1 | 1 | Low | No | GAS | 100 | 1 | 4 | 3 | Child |
| 11,M | 2,H | SE | V | 1 | 1 | Low | No | GAS | 200 | 5 | 5 | 0 | Child |
| 18,F | 3,Q | SE | V | 1 | 2 | Low | No | GASs | 400 | 3 | 5 | 2 | Child |
| **Botulinum Toxin Injections with Medical Clowning** | | | | | | |  |  |  |  |  |  |  |
| 2,M | 4,Q | SE | V | 1 | 3 | Up, Low | No | HAMs, PT | 100 | 4 | 3 | -1 | Parent |
| 2,F | 3,D | SE | NV | 1 | 4 | Low | No | ADDs HAMs | 160 | 5 | 3.5 | -1.5 | Parent |
| 3,M | 3,Q | SE | NV | 1 | 5 | Up, Low | No | ADDs HAMs PT | 240 | 4 | 5 | 1 | Parent |
| 3,M | 3,H | SE | V | 2 | 2 | Up, Low | No | R HAM,PT | 60 | 4 | 5 | 1 | Parent |
| 4,M | 3,Q | SE | NV | 1 | 6 | Low | Yes | GASs TPs HAMs | 200 | 3 | 4 | 1 | Parent |
| 4,M | 2,D | SE | V | 2 | 4 | Low | No | GASs HAMs | 300 | 3 | 3 | 0 | Parent |
| 5,F | 3,D | SE | V | 1 | 4 | Low | No | HAMs,GASs | 200 | 2 | 1 | -1 | Parent |
| 5,F | 3,D | SE | V | 1 | 4 | Low | No | HAMs,GASs | 200 | 3 | 4 | 1 | Parent |
| 6,M | 2,D | SE | V | 1 | 2 | Low | Yes | GAS TP | 160 | 1 | 1 | 0 | Child |
| 7,M | 3,D | SE | V | 4 | 3 | Low | Yes | GASs TP | 130 | 4 | 1 | -3 | Child |
| 8,F | 4,D | SE | V | 4 | 2 | Low | No | HAMs | 260 | 5 | 1 | -4 | Child |
| 8,M | 2,D | N | V | 1 | 2 | Up, Low | No | PT GAS | 150 | 5 | 2 | -3 | Child |
| 8,M | 2,H | N | V | 1 | 3 | Up, Low | Yes | PT,TIB POST, GAS | 200 | 1 | 5 | 4 | Child |
| 9,F | 2,H | N | V | 4 | 2 | Up | Yes | FCU, ADD POL | 50 | 3 | 3 | 0 | Child |
| 10,M | 5,Q | SE | NV | 1 | 6 | Up | No | BIC BR BRACH FCU FCR FDP | 190 | 3.5 | 3.5 | 0 | Child |
| 12,M | 2,D | SE | V | 1 | 1 | Low | No | GAS | 200 | 1 | 1 | 0 | Child |
| 14,F | 3,H | N | V | 1 | 8 | Up, Low | No | Bic, BR, PT,FCU,HAM, GASs | 300 | 3 | 4 | 1 | Child |
| 16,M | 4,Q | SE | V | 2 | 2 | Low | No | GASs | 400 | 5 | 2.5 | -2.5 | Child |
| 16,M | 2,D | N | V | 1 | 2 | Low | No | HAMs | 400 | 3 | 2 | -1 | Child |

Pleagia: H=hemiplegia, D=diplegia, Q=quadriplegia, T=triplegia; School: SE= special education; MS= main stream; Comm..= communication skills: NV= non verbal, V= verbal; Inject #= injection number: 1= first etc.; Limb (injected): Low=lower, Up=Upper; IU=total amount; Muscles: Bic=Biceps, BR bracioradialis, Bra=brachialis, Gas= gastrocnemius, TP= tibialis posterior, PT= Pronotor Teres; Ham=hamstrings, ADD=Adductor, FCU=Flexor Carpi Ulnaris, FCR= Flexor Carpi Radialis, FDP= Flexor Dgitorium Perfunnus, ADD POL = Addctor Pollicis Brevis. (s)=bilateral injection

**TABLE 2: Tests of Between-Subjects Effects for Pain after Injections**

| **Parameter** | **Type III Sum of Squares** | **df** | **Mean Square** | **F** | **Sig.** |
| --- | --- | --- | --- | --- | --- |
| Corrected Model | 37.876^a^ | 11 | 3.443 | 3.079 | 0.014 |
| Intercept | 0.538 | 1 | 0.538 | 0.481 | 0.496 |
| Age | 1.229 | 1 | 1.229 | 1.099 | 0.307 |
| Gender | 0.012 | 1 | 0.012 | 0.011 | 0.917 |
| GMFCS | 3.979 | 1 | 3.979 | 3.558 | 0.074 |
| School attendance | 1.476 | 1 | 1.476 | 1.319 | 0.264 |
| Communication skills | 2.848 | 1 | 2.848 | 2.546 | 0.126 |
| # of injection | 0.001 | 1 | 0.001 | 0.001 | 0.974 |
| Sensitive sites | 0.661 | 1 | 0.661 | 0.591 | 0.451 |
| # muscles | 1.689 | 1 | 1.689 | 1.511 | 0.233 |
| Upper Limb | 7.219 | 1 | 7.219 | 6.455 | 0.019 |
| Report (parent/child) | 1.694 | 1 | 1.694 | 1.514 | 0.233 |
| Clown care* | 23.71 | 1 | 23.71 | 21.202 | 0.000 |
| Error | 22.366 | 20 | 1.118 |  |  |
| Total | 455.75 | 32 |  |  |  |
| Corrected Total | 60.242 | 31 |  |  |  |

^a^ R Squared = .629 (Adjusted R Squared = .425)

**Legends**

**Figure 1: Medical clowning techniques used during BTX injections**

Before: Clown engaging and distracting the anxious child who pays attention to the scene in front of him. During the injection (in front): Clown and child in a secluded atmosphere at the other end of the table; parent watching with a smile. After: Child is laughing leaving the room empowered.

**Figure 2: Visual Analogue Scale pain rating clown care versus control**

There was no difference in pain anticipation before BTX injection with clown care (black bars) versus control (white bars). After the procedure there was less pain among the study group (black bars) then in controls (white).
